# Supplementary material for: Population Dynamics of Aedes aegypti and Aedes albopictus in Two Rural Villages in Southern Mexico: Baseline Data for an Evaluation of the Sterile Insect Technique
Source: Insects. 2021 Jan 11;12(1):58. doi: 10.3390/insects12010058 (PMC7827525; doi:10.3390/insects12010058)
Supplement: Supplementary file 1 [file insects-12-00058-s001.pdf]

SUPPLEMENTAL MATERIAL

Table S1. Data of the 4-week periods and dates of the dry and rainy seasons during 3-years of sampling at the Hidalgo and Río Florido villages. The rainy season was taken to have started when precipitation exceeded 100 mm during a 4-week period, and to have ceased when precipitation fell below this level.

	Dry season		Rainy season	
Sampling year	4-week periods	Date	4-week periods	Date
2016	1 - 4	3 January to 23 April, 2016	5 - 12	24 April to 3 December, 2016
2016-2017	13 - 3	4 December, 2016 to 25 March, 2017	4 - 11	26 March to 4 November, 2017
2017-2018	12 - 3	5 November, 2017 to 24 March, 2018	4 - 12	25 March to 1 December, 2018

Table S2. Mixed model analyses of deviance with type II Wald χ^2 tests (negative binomial responses)

I: Egg counts from ovitraps inside and outside of houses within villages

**Source**	χ^2	**d.f.**	**P**
Year (2016 – 2018)	148.9013	2	**< 2.2e-16**
Season (rainy, dry)	69.2567	1	**2.2e-16**
Village (Hidalgo, Río Florido)	1.7546	1	0.185294
Location (indoor, outdoor)	7.4052	1	**0.006504**
Season*location	1.0361	1	0.308739
Season*village	0.5898	1	0.442505
Season*location*village	0.2698	1	0.603495

P values in bold type are significant (P<0.05).

II. Estimated abundance of *Ae. aegypti* and *Ae. albopictus* in samples from within villages

**Source**	χ^2	**d.f.**	**P**
Year (2016 – 2018)	541.3414	2	**< 2.2e-16**
Season (rainy, dry)	44.1734	1	**3.005e-11**
Village (Hidalgo, Río Florido)	0.4235	1	0.51521
Location (indoor, outdoor)	0.0319	1	0.85817
Species (Ae. aegypti, Ae. albopictus)	31.6260	1	**1.869e-08**
Season*location	0.0134	1	0.90773
Season*species	0.0055	1	0.94082
Location*species	44.4385	1	**2.625e-11**
Season*village	0.2231	1	0.63671
Location*village	4.5333	1	**0.03324**
Species*village	137.9253	1	**< 2.2e-16**
Season*location*species	2.1050	1	0.14681
Season*location*village	0.0011	1	0.97335
Season*species*village	0.1680	1	0.68191
Location*species*village	6.4034	1	**0.01139**
Season*location*species*village	0.0353	1	0.85105

P values in bold type are significant (P<0.05)

III. Egg counts from ovitraps placed along transects at a distance of 0, 50 or 100 m from the edge of each village

**Source**	χ^2	**d.f.**	**P**
Year (2016 – 2018)	77.5670	2	**< 2.2e-16**
Season (rainy, dry)	185.0430	1	**< 2.2e-16**
Village (Hidalgo, Río Florido)	6.1401	1	**0.0132150**
Location (0, 50, 100 m)	54.6687	2	**1.345e-12**
Village*location	15.9627	2	**0.0003418**
Season*village	0.0182	1	0.8925735
Season*location	4.4939	2	0.1057225
Village*season*location	2.8793	2	0.2370120

P values in bold type are significant (P<0.05)

Table S3. Average (± SE) air temperature, relative humidity and temperature of water from ovitraps at the moment of sampling. Ovitraps were placed inside or outside houses in the dry and rainy seasons in the villages of Río Florido and Hidalgo during a 3-year study. Rainfall was recorded at a nearby weather station.

		**Río Florido**			**Hidalgo**			
Year and season	Location	Air Temp (°C)	RH (%)	Water Temp (°C)	Air Temp (°C)	RH (%)	Water Temp (°C)	Total Rainfall (mm)
2016 Dry	Indoor	34.2 ± 0.2	43.8 ± 0.6	26.9 ± 0.1	31.7 ± 0.1	53.5 ± 0.7	27.5 ± 0.1	9
	Outdoor	34.1 ± 0.1	45.1 ± 0.6	27.6 ± 0.1	31.1 ± 0.1	59.9 ± 0.4	26.9 ± 0.1	
2016 Rainy	Indoor	33.0 ± 0.1	61.0 ± 0.6	28.2 ± 0.1	32.0 ± 0.1	63.8 ± 0.5	27.7 ± 0.1	2,908
	Outdoor	32.8 ± 0.1	61.5 ± 0.6	28.2 ± 0.1	32.0 ± 0.1	64.0 ± 0.4	27.5 ± 0.1	
2016-2017 Dry	Indoor	32.4 ± 0.2	55.6 ± 0.8	26.6 ± 0.1	30.9 ± 0.1	59.4 ± 0.7	26.4 ± 0.1	52
	Outdoor	32.4 ± 0.2	55.4 ± 0.8	26.7 ± 0.1	30.8 ± 0.1	59.9 ± 0.6	26.1 ± 0.1	
2017 Rainy	Indoor	32.5 ± 0.1	65.9 ± 0.6	27.9 ± 0.1	29.4 ± 0.1	82.5 ± 0.5	28.2 ± 0.1	2,740
	Outdoor	32.4 ± 0.1	66.3 ± 0.6	28.0 ± 0.1	29.4 ± 0.1	80.4 ± 0.4	27.7 ± 0.1	
2017-2018 Dry	Indoor	32.2± 0.2	56.1 ± 0.7	27.9 ± 0.1	29.0 ± 0.1	75.3 ± 0.7	27.1 ± 0.1	25
	Outdoor	32.2 ± 0.2	56.6 ± 0.7	28.2 ± 0.1	29.0 ± 0.1	74.6 ± 0.5	26.9 ± 0.1	
2018 Rainy	Indoor	32.0 ± 0.1	68.4 ± 0.6	28.3 ± 0.1	29.1 ± 0.1	88.0 ± 0.3	27.7 ± 0.1	2,837
	Outdoor	32.1 ± 0.1	68.4 ± 0.5	28.3 ± 0.1	29.2 ± 0.1	86.2 ± 0.3	27.4 ± 0.1	

Note: Rainy season was end of March-April to October-November, dry season was November to April

Table S4. Means (± EE) of air temperature, relative humidity and water temperature from ovitraps at the moment of sampling. Ovitraps were located at 0, 50 and 100 m points in five transects from the edge of the villages of Río Florido and Hidalgo during the dry and rainy seasons of a 3-year study. The data for the road transect between the villages were taken between 13 December 2017 and 28 November 2018.

		Río Florido			Hidalgo		
Year and season	Distance from edge of village	Air Temp (°C)	RH (%)	Water Temp (°C)	Air Temp (°C)	RH (%)	Water Temp (°C)
2016 Dry	0 m	31.2 ± 0.2	58.1 ± 0.9	24.4 ± 0.1	32.2 ± 0.3	55.3 ± 1.4	25.5 ± 0.2
	50 m	30.9 ± 0.2	58.9 ± 0.9	24.7 ± 0.1	32.3 ± 0.3	55.6 ± 1.5	26.0 ± 0.2
	100 m	31.0 ± 0.2	59.8 ± 0.9	24.8 ± 0.1	32.2 ± 0.3	55.8 ± 1.6	25.9 ± 0.3
2016 Rainy	0 m	29.3 ± 0.1	77.6 ± 0.7	26.1 ± 0.1	31.8 ± 0.2	65.5 ± 0.9	26.4 ± 0.2
	50 m	29.1 ± 0.1	78.9 ± 0.6	26.1 ± 0.1	31.6 ± 0.2	65.8 ± 0.9	26.4 ± 0.1
	100 m	29.1 ± 0.1	80.2 ± 0.6	26.1 ± 0.1	31.3 ± 0.2	67.7 ± 0.9	26.5 ± 0.2
2016-2017 Dry	0 m	29.1 ± 0.2	70.9 ± 1.3	24.3 ± 0.1	30.8 ± 0.3	62.3 ± 1.1	25.0 ± 0.2
	50 m	28.9 ± 0.2	70.8 ± 1.3	24.1 ± 0.1	30.5 ± 0.3	62.9 ± 1.2	24.9 ± 0.2
	100 m	29.1 ± 0.2	72.2 ± 1.2	24.1 ± 0.1	30.3 ± 0.3	64.3 ± 1.2	25.3 ± 0.2
2017 Rainy	0 m	29.1 ± 0.1	78.7 ± 0.7	26.0 ± 0.1	29.5 ± 0.2	81.3 ± 0.7	27.2 ± 0.1
	50 m	29.1 ± 0.1	78.9 ± 0.7	25.8 ± 0.1	29.6 ± 0.2	81.3 ± 0.8	27.1 ± 0.1
	100 m	29.3 ± 0.2	79.1 ± 0.7	26.0 ± 0.1	29.3 ± 0.2	82.7 ± 0.7	27.3 ± 0.1
2017-2018 Dry	0 m	28.5 ± 0.2	70.5 ± 0.8	25.3 ± 0.1	29.4 ± 0.3	75.1 ± 1.0	25.8 ± 0.2
	50 m	28.4 ± 0.2	71.5 ± 0.9	25.1 ± 0.1	29.1 ± 0.3	76.6 ± 0.9	26.1 ± 0.2
	100 m	28.5 ± 0.2	72.0 ± 0.8	25.1 ± 0.1	29.1 ± 0.3	77.6 ± 0.9	26.0 ± 0.2
2018 Rainy	0 m	29.2 ± 0.1	78.3 ± 0.6	26.4 ± 0.1	29.7 ± 0.2	85.8 ± 0.6	26.9 ± 0.2
	50 m	29.0 ± 0.1	79.6 ± 0.6	26.3 ± 0.1	29.6 ± 0.2	86.9 ± 0.4	27.1 ± 0.2
	100 m	29.0 ± 0.1	80.1 ± 0.5	26.3 ± 0.1	29.3 ± 0.2	88.1 ± 0.4	27.0 ± 0.1
2017-2018 Dry	Transect along road between villages						
		36.3 ± 0.2	45.1 ± 1.0	30.1 ± 0.2			
2018 Rainy		34.8 ± 0.1	62.9 ± 0.8	29.3 ± 0.1			

Table S5. Total number of *Aedes* spp. eggs collected in ovitraps in the surrounding transects Río Florido, number of eggs tested in laboratory, and number and prevalence of *Ae. aegypti* and *Ae. albopictus* determined by laboratory rearing of samples between January 2016 to November 2018.

				Ae. aegypti	***Ae. albopictus***
**Year and season of sample**	**Location (distance along transect)**	**Total eggs collected in ovitraps**	**Total eggs tested in laboratory**	**Number of larvae reared (%)**	**Number of larvae reared (%)**
2016 Dry	0 m	1,583	321	10 (7.7)	120 (92.3)
	50 m	858	355	15 (7.7)	181 (92.3)
	100 m	386	280	8 (5.6)	135 (94.4)
2016 Rainy	0 m	7,729	3,155	46 (4.5)	970 (85.5)
	50 m	4,227	2,333	11 (1.1)	983 (98.9)
	100 m	3,705	2,092	10 (0.9)	1,059 (99.1)
2016-2017 Dry	0 m	861	449	1 (0.6)	158 (99.4)
	50 m	747	434	0 (0.0)	181 (100)
	100 m	769	364	1 (0.6)	157 (99.4)
2017 Rainy	0 m	9,427	3,925	17 (0.7)	2,468 (99.3)
	50 m	8,457	3,728	6 (0.2)	2,500 (99.8)
	100 m	5,333	2,563	1 (0.1)	1,409 (99.9)
2017-2018 Dry	0 m	3,417	2,202	2 (0.2)	1,153 (99.8)
	50 m	2,852	1,922	0 (0.0)	1,113 (100)
	100 m	2,235	1,210	0 (0.0)	694 (100)
2018 Rainy	0 m	10,090	3,517	16 (0.7)	2,224 (99.3)
	50 m	13,411	4,622	41 (1.4)	2,856 (98.6)
	100 m	7,960	3,063	2 (0.1)	1,635 (99.9)
	**Totals**	**84,047**	**36,535**	**187**	**19,996**

Table S6. Total number of *Aedes* spp. eggs collected in ovitraps in the surrounding transects Hidalgo, number of eggs tested in laboratory, and number and prevalence of *Ae. aegypti* and *Ae. albopictus* determined by laboratory rearing of samples between January 2016 to November 2018.

				Ae. aegypti	***Ae. albopictus***
**Year and season of sample**	**Location (distance along transect)**	**Total eggs collected in ovitraps**	**Total eggs tested in laboratory**	**Number of larvae reared (%)**	**Number of larvae reared (%)**
2016 Dry	0 m	970	473	16 (7.6)	195 (92.4)
	50 m	458	299	30 (17.4)	142 (82.6)
	100 m	406	184	15 (17.2)	72 (82.8)
2016 Rainy	0 m	5,147	1,792	46 (6.9)	625 (93.1)
	50 m	2,533	1,092	3 (0.6)	480 (99.4)
	100 m	3,723	1,384	49 (8.4)	535 (91.6)
2016-2017 Dry	0 m	1,605	887	54 (13.8)	336 (86.2)
	50 m	778	482	0 (0.0)	254 (100)
	100 m	885	590	0 (0.0)	367 (100)
2017 Rainy	0 m	8,357	3,614	228 (10.8)	1,884 (89.2)
	50 m	3,606	1,914	26 (3.3)	765 (96.7)
	100 m	4,069	2,151	25 (3.3)	724 (96.7)
2017-2018 Dry	0 m	4,292	1,856	60 (6.0)	946 (94.0)
	50 m	963	767	0 (0.0)	374 (100)
	100 m	1,204	833	0 (0.0)	323 (100)
2018 Rainy	0 m	10,643	4,070	153 (7.2)	1,980 (92.8)
	50 m	8,247	2,518	16 (1.9)	825 (98.1)
	100 m	9,656	2,443	10 (0.8)	1,199 (99.2)
	**Totals**	**67,542**	**27,349**	**731**	**12,026**

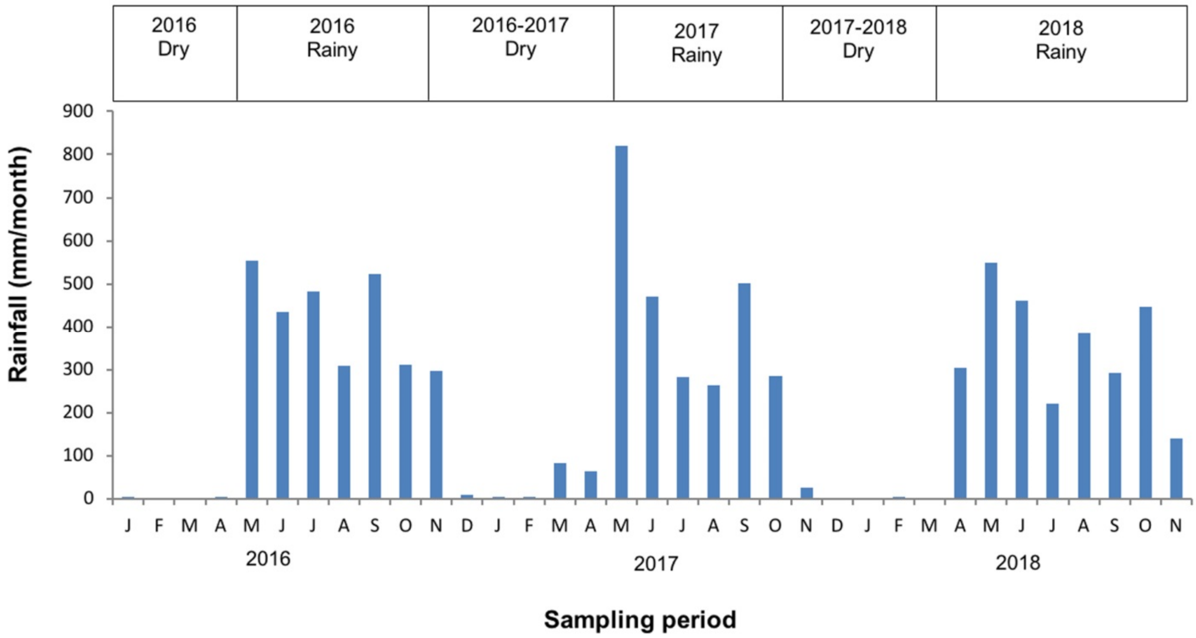

Figure S1. Monthly rainfall recorded during three years of sampling at the Hidalgo and Río Florido villages.

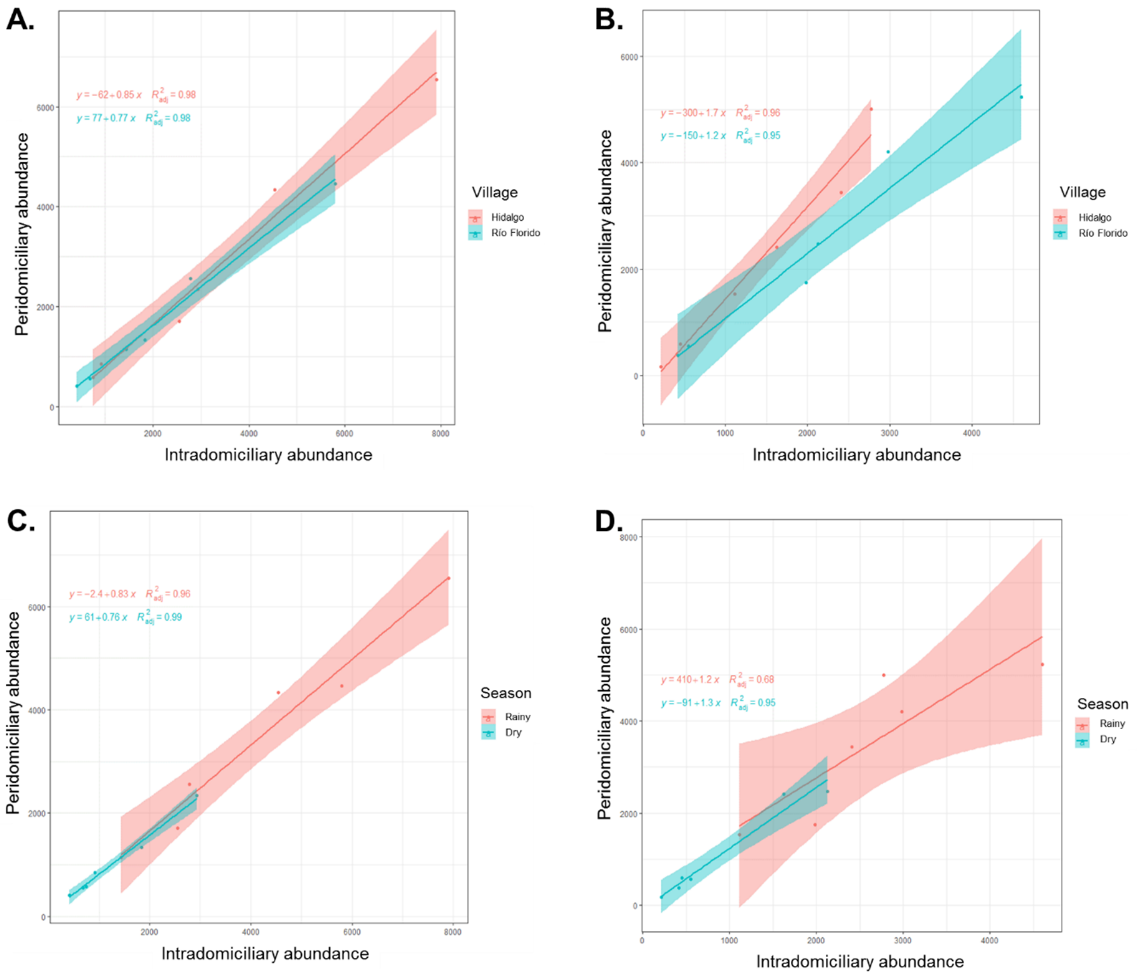

Figure S2. Correlations between intradomiciliary and peridomiciliary seasonal estimated abundance of adults of (A) *Ae. aegypti* and (B) *Ae. albopictus* for each village, and (C) *Ae. aegypti* and (D) *Ae. albopictus* for the rainy and dry seasons.
